# Changes in physical activity in people with idiopathic pulmonary fibrosis before and after virtual pulmonary rehabilitation: a feasibility study

**DOI:** 10.1186/s12890-024-03030-9

**Published:** 2024-05-02

**Authors:** Orlagh O’Shea, Grainne Murphy, Lynn Fox, Katherine M.A. O’Reilly

**Affiliations:** 1 School of Physiotherapy, Royal College of Surgeons in Ireland, Dublin, Ireland; 2 Department of Physiotherapy, Mater Misericordiae University Hospital, Dublin, Ireland; 3 Department of Respiratory Medicine, Mater Misericordiae University Hospital, Dublin, Ireland; 4 School of Medicine, University College Dublin, Dublin, Ireland

**Keywords:** Pulmonary rehabilitation, Virtual Pulmonary Rehabilitation, Idiopathic pulmonary, Fibrosis, Physical activity

## Abstract

**Background:**

Pulmonary rehabilitation (PR) is recommended for the treatment of people with idiopathic pulmonary fibrosis (IPF). Physical activity is an important health behaviour, closely linked to survival in people with IPF. Little is known about the impact of virtual (V) PR on physical activity in people with IPF.

**Objective:**

To explore the feasibility of conducting a trial to explore effect of virtual PR on objectively measured physical activity in people with IPF.

**Methods:**

All patients with a diagnosis of IPF in a stable phase of the disease were invited to participate in VPR: a 10 week exercise programme delivered twice-weekly for one hour. Data were collected at baseline (BL) and post VPR (10 weeks): Kings Brief Interstitial Lung Disease (K-BILD), Exercise capacity (6-minute walk test (6MWT) or 1-minute sit-to-stand (STS)) and Physical Activity. Physical activity was measured with a triaxial accelerometer for seven days. Screening, recruitment, adherence and safety data were collected.

**Results:**

68 people were screened for this study. *N* = 16 participants were recruited to the study. There was one dropout. *N* = 15 completed VPR. All results reported in mean (standard deviation) (SD). Participants attended 18.1(2.0) of the 20 sessions. No adverse events were detected. The mean age of participants was 71.5(11.5) years, range: 47–95 years; 7 M:9 F. Mean (SD) FEV_1_ 2.3(0.3)L, FVC 2.8(0.7)L. No statistically significant changes were observed in outcome measures apart from exercise capacity. Light physical activity increased from 152(69.4) minutes per day (*n* = 16) to 161.9(88.7) minutes per day (*n* = 14), mean change (SD) (CI) *p-value*: 9.9 (39.8) [-12.3 to 30.9] *p =* 0.4. Moderate-to-vigorous physical activity increased from 19.1(18.6) minutes per day (*n* = 16) to 25.7(28.3) minutes per day (*n* = 14), mean change (SD) (CI) *p-value*: 6.7 (15.5) [-2.1 to 15.1] *p* = 0.1. Step count increased from 3838(2847) steps per day (*n* = 16) to 4537(3748) steps per day (*n* = 14), mean change (SD) (CI) *p-value*: 738 (1916) [-419.3 to 1734.6] *p* = 0.2. K-BILD (*n* = 15) increased from 55.1(7.4) at BL to 55.7(7.9) post VPR mean change (SD) [95% confidence interval] (CI) *p-value*: 1.7(6.5) [-1.7 to 5.3], *p* = 0.3. 6MWT (*n* = 5) increased from 361.5(127.1) to 452.2(136.1) meters, mean change (SD) (CI) *p-value*: 63.7 (48.2) [-3.8 to 123.6], *p* = 0.04 and 1-minute STS increased from 17.6(3.0) (*n* = 11) to 23.7(6.3) (*n* = 10), mean change (SD) (CI) *p*-*value* 5.8 (4.6) [2.6 to 9.1], *p* = 0.003.

**Conclusion:**

VPR can improve physical activity in people with IPF. A number of important feasibility issues included recruitment, retention, adherence and safety have been reported which are crucial for future research in this area. A fully powered trial is needed to determine the response of people with IPF to PR with regard to physical activity.

**Supplementary Information:**

The online version contains supplementary material available at 10.1186/s12890-024-03030-9.

## Background

Idiopathic pulmonary fibrosis (IPF) is a chronic and progressive lung disease of unknown aetiology that is associated with significant morbidity and mortality [1]. IPF is characterised by progressive scarring of the lung parenchyma leading to distorted lung architecture and progressive deterioration of lung function and impaired gas exchange. Patients experience increasing dyspnoea and frequently develop hypoxemia [2, 3]. Consequently, people with IPF reduce their levels of physical activity [4]. Lower levels of physical activity are associated with worse physiological function [5]. In general low levels of physical activity are detrimental to individuals’ health, leading to muscle wasting and fatigue [6], ultimately leading to a significantly worse survival for people with IPF [5].

Pulmonary rehabilitation (PR) is a non-pharmacological exercise and education based programme recommended for people with IPF both nationally in Ireland and internationally [7, 8]. PR can increase exercise capacity and HRQoL in people with IPF [9] and Cox et al. reported that virtual PR (VPR) can achieve outcomes similar to those of traditional centre-based PR, with no safety issues [10]. However, the review by Cox et al. is limited with regard to evidence for IPF, the studies included in this review only involved patients with COPD [10]. There is therefore a limited evidence based for VPR for people with IPF. Furthermore, the impact of PR on physical activity in people with IPF is not well documented. Only two studies have explored changes in physical activity following PR specifically in people with IPF [11, 12]. However, both these studies used self-report measures of physical activity [11, 12]. Self-report measures of physical activity have been reported to have multiple sources of error, including recall bias and an caution is advised when using self-report measures to evaluate an intervention [13, 14]. Ng et al. conducted a systematic review on changes in physical activity following exercise interventions in COPD [15]. This review recommended that all future studies employ triaxial accelerometry to accurately assess the impact of exercise interventions on physical activity levels [15]. To the authors’ knowledge to date no study has explored the impact of VPR on physical activity as measured with a trixial accelerometer in people with IPF. Therefore, the aim of this study is to explore the feasibility of conducting a trial to explore effect of VPR on objectively measured physical activity in people with IPF.

## Methods

This reporting of this feasibility study follows the CONSORT 2010 statement: extension to randomised pilot and feasibility trials [16] with further guidance from Lancaster and Thabane 2019 [17]. Ethical approval was obtained from the Mater Misericordiae University Hospital, Research Ethics Committee. Institutional review board reference: 1/378/2111.

### Participant recruitment

We aimed to recruit 30 individuals to this feasibility trial [18]. All individuals with IPF who were referred to PR were screened for eligibility by the author GM. Those meeting the inclusion criteria for PR: functionally limited by breathlessness and in stable phase of IPF were invited to participate by GM during a telephone consultation. A stable phase of IPF was defined as patients who do not have rapidly escalating symptoms or rapidly increasing oxygen needs. Patients needed to provide their own device (e.g. tablet or laptop) and internet access to participate in the programme. Unfortunately, due to a lack funding we were not able to provide participants with devices or internet access to enable participation. Written informed consent was obtained by post. Patients referred for palliative care were excluded from the programme as patients currently under the care of palliative care have access to a dedicated PR programme run within the hospice and support from hospice based physiotherapy. Those wishing to participate in the VPR but not research aspect were not excluded from the programme.

## Intervention - pulmonary rehabilitation

All participants underwent a 10 week VPR programme. There was no in-person PR being delivered at this time due to the COVID-19 pandemic. The VPR was delivered by a senior physiotherapist (GM) with 27 years experience as a senior respiratory physiotherapist delivering PR programmes. This programme was specifically designed for people with interstitial lung diseases (ILD) including IPF. The programme consisted of twice-weekly, one hour group exercise classes for 10 weeks. There was a maximum of six participants in a group. The programme was delivered via the Salaso platform (Salaso Health Solutions, Ireland). Salaso is a video conferencing platform similar to Zoom.

The VPR exercise classes participants completed in a series of exercises including a warm up, upper and lower limb strengthening exercises (e.g. squats, shoulder press) and aerobic exercises (e.g. marching on the spot, heel taps). The participants participated in interval based training whereby the exercised for one minute and then had a 30 s rest period. Over the course of the 10 weeks this one minute was gradually increased to 1.5 min of exercise, the rest period remained the same. Participants were advised to exercise at an intensity of BORG 4 during this period. Individual progressions were made whereby participants progressed to using weights when they felt able. These weights which were obtained by participants themselves, they either acquired dumbbells or used household items (e.g. a 500 ml bottle of water or a tin of beans). No further equipment was used during the exercise classes. Furthermore participants could progress individually by increasing the number of repetitions they were completing during the time for exercise. Oxygen saturations, heart rate and BORG are documented at the beginning of the class. They were reassessed at the end of every bout of exercise participants’ oxygen saturation, heart rate and exertion level. Furthermore, prior to participation all participants had to provide an emergency contact of an individual who would be readily available in the event of an adverse event as safety measure.

Two formal education sessions (45 min in duration) were delivered: the benefits of exercise (including a home exercise programme) and Conservation of Energy. Breathing control methods were taught throughout each class and relaxation was performed at the end of each class. Individual consultations with the facilitator were facilitated on an informal basis before or after the class at the request of participants.

### Data collection

Demographic data including age, gender and pulmonary function tests (FEV_1_, (forced expiratory volume in the first second) FVC, (forced vital capacity) TLCO (transfer capacity of lung for carbon monoxide) were collected at baseline. Health related quality of life (HRQoL), exercise capacity tests and physical activity measurements were collected at baseline and post intervention. Adherence to PR was also recorded.

HRQoL was measured using The Kings Brief Interstitial Lung Disease (K-BILD). K-BILD is a health status questionnaire developed and validated specifically for patients with ILD [19]. The K-BILD contains 15 items that measures health status in three domains: (1) psychological, (2) breathlessness and activities and (3) chest symptoms. The K-BILD is scored on a scale of 0-100, with 100 representing the best possible health. The minimal clinical important difference for the K-BILD is 3.9 points [19]. The MCID estimates for KBILD-Psychological, KBILD-Breathlessness and activities, and K-BILD Chest symptoms were 5.4, 4.4 and 9.8 points, respectively [20].

Exercise capacity: due to COVID-19 related restrictions at various times throughout the study period, different exercise capacity tests were used. Details of the COVID-19 restrictions throughout the study period are summarised in the e-supplement. Including the 6 min walk test (6MWT) [21] which was conducted in person or the 1-minute sit-to-stand (STS) conducted remotely [22, 23]. For the one-minute STS participants were instructed to use a stable kitchen or dining room chair.

Physical activity was measured using a triaxial accelerometer (Actigraph gt3x) (Actigraph LLC; Pensacola, FL) which was worn around the waist for seven consecutive days during waking hours. Participants wore the Actigraph before commencing PR and within one week of finishing PR. Further details regarding the how the Actigraph was worn are available in Table 1.

**Table 1:** Reporting checklist for accelerometer based physical activity measurement

Items	Comments
Name of activity monitor	Actigraph
Model of activity monitor	wGT3X
Epoch length	Recorded in 1 s epoch and then reintegrated into 15 s epoch for analysis
Type of sensors	Acceleration and ambient light sensors
Location of activity monitor	Waist worn
Side of activity monitor	Right side
Distribution way of activity monitor	In and person and by post
Number of participants enrolled at start of study receiving accelerometers	16
Days of data collected (number of days you instructed the participants to wear the monitor)	Participants were instructed to wear the monitor for 7 days
Hours of data collected per day (number of hours you instructed participants to wear the monitor)	Participants were instructed to wear the device for all waking hours. But not in the shower
Weekday or weekend day requirement	No requirement
Criteria for non-wearing of activity monitors (how you defined non-wearing time)	Choi algorithm - The algorithm “Choi” defines non-wear times as periods of consecutive 0-counts of a certain duration. This duration is defined as “minimum length of non-wear times”. The default setting by the manufacturer is 90 min.
How many hours of activity monitor data needed to be considered a valid day	10 h per day
Number of valid days of activity monitor data needed to be included in analysis	5 days per week
Other rules for excluding from analysis (e.g., at least 3 weekdays and 1 weekend data are required)	No other rules
Physical activity outcomes (or metrics) used	Light, moderate to vigorous and step count
Number of people not meeting wear-time criteria and excluded from analysis	All participants met wear time criteria.

### Data analysis

The Actigraph data were analysed in Actilife version 6. See Table 1 Actigraph data reporting checklist [24]. All data were entered in STATA (StataCorp, United States of America) 17.0 and analysed for descriptive statistics. Data were tested for normality and paired student t test were performed to explore changes.

## Results

### Participants

Sixty-eight participants were referred to the programme over a 33 month study period (August 2020-April 2022). The mean (standard deviation) (SD) age of participants was 71.5 (11.5) years, range: 47–95 years. There were seven male and nine female participants in this study. Four participants were home oxygen users (*n* = 1 on 4 L/min, *n* = 2 on 6 L/min and *n* = 1 on 10 L/min). Ten participants were on oral antifibrotic medications. Full demographic details are available in Table 2. Since completion of the study five participants have died, these participants died between 1 and 22 months after finishing the programme, a mean survival time of 12 months was observed for these participants.

### Feasibility Data

**Fig. 1:**
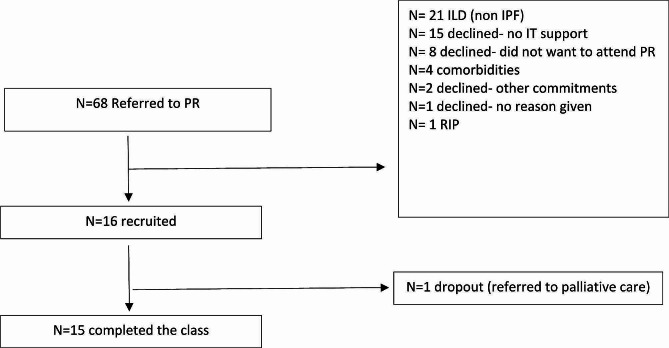
CONSORT flow diagram

Sixteen were recruited, see Fig. 1 for full screening details. Fifteen participants completed the VPR, *n* = 1 dropped out as they experienced an acute worsening of their disease. We were unable to collect post PR physical activity outcomes for one participant due to COVID-19 related complications. The mean (SD) adherence to the programme was 18.1 (2.0) classes. All participants, had >/ 80% adherence except for one participant who only adhered to 65% of classes due to illness. No adverse events were reported.

#### HRQol and Exercse Capacity

Results for HRQoL and exercise capacity are available in Table 3.

### Physical activity

All participants met the wear time data rules of a minimum of ten hours on five days. The mean (SD) wear time across all the data was 831 (76) minutes over a mean of 7 (0.4) days.

**Fig. 2:**
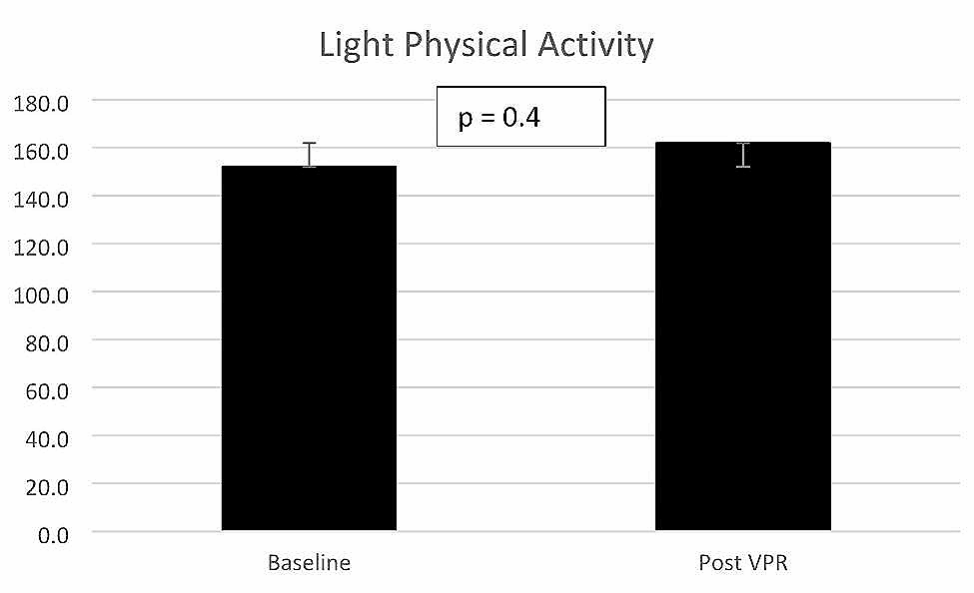
Changes in minutes of mean daily light physical activity (1.6–2.9 METS) with standard error bars * VPR = virtual pulmonary rehabilitation

**Fig. 3:**
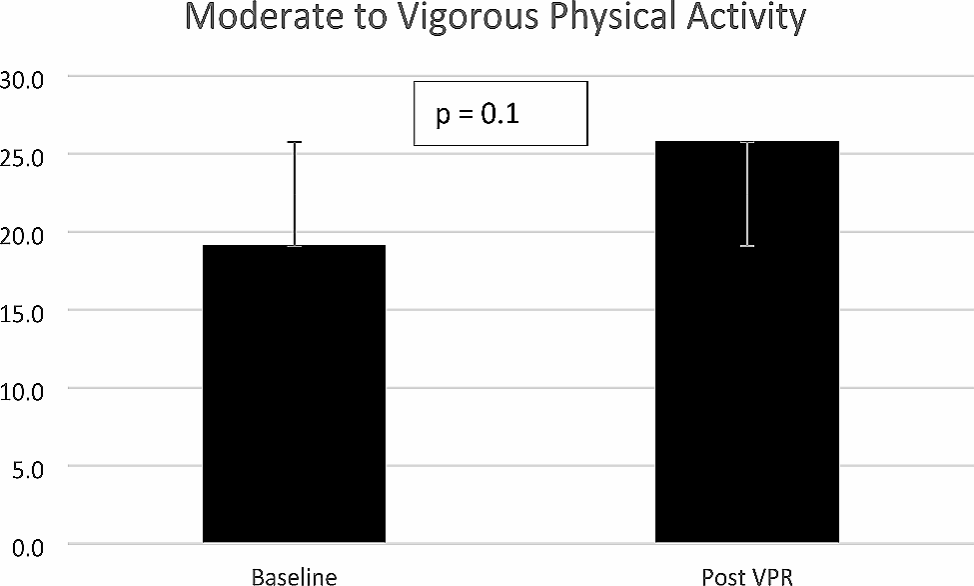
Changes in minutes mean daily moderate to vigorous physical activity (3 ->6 METS) with standard error bars * VPR = virtual pulmonary rehabilitation

**Fig. 4:**
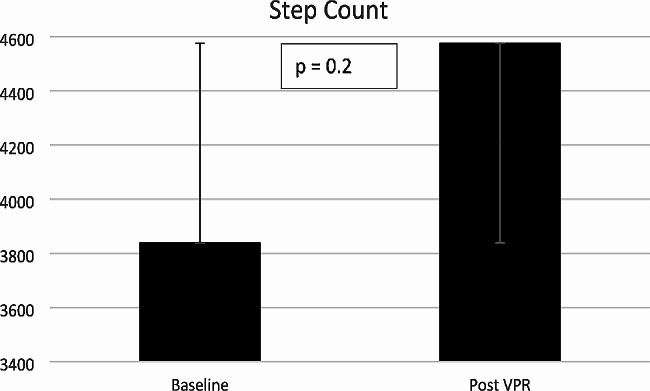
Changes in mean daily step count with standard error bars * VPR = virtual pulmonary rehabilitation

Light physical activity increased from mean (SD) 152 (69.4) minutes per day at baseline to 161.9 (88.7) minutes post VPR (mean (SD) difference 9.9 (38.8) minutes per day. Light physical activity is defined as any activity between > 1.5–3 metabolic equivalents (METS). Moderate to vigorous physical activity (MVPA) increased from mean (SD) 19.1 (18.6) minutes per day to 25.7 (28.3) minutes post VPR (mean (SD) difference 6.7 (15.5) minutes per day. Moderate physical activity is defined as any activity > 3 METS. Step count increased from mean (SD) 3838 (2847) steps per day at baseline to 4576 (3748) steps post VPR per day (mean (SD) difference 738 (1916) steps). None of these improvements in physical activity were statistically significant. Table 3 for details on mean difference, 95% confidence intervals and p-values. Figures 2, 3 and 4 demonstrate the variations across individual changes in physical activity with SD error bars included in the line graphs.

**Table 2:** Demographic information for participants

Parameter	Mean (SD) Range or Frequency
Gender	7 male 9 female
Age	71.4 (11.5) 47–95 years
Lung function FEV_1_* (Available for *n* = 13)	2.3 (0.3)L (1.8–2.8)L
FEV_1_*% (Available for *n* = 14)	92.1 (18.9)% (67.4–117.9)%
FVC** (Available for *n* = 14)	2.8 (0.7)L (0.07–3.6)L
FVC%** (Available for *n* = 15)	85.2 (19.2)% (35.0- 113.0)%
FEV_1_/FVC (Available for *n* = 13)	0.8 (0.0) (0.7–0.9)
TLCO*** (Available for *n* = 11)	4.5 (1.3)L (2.3–6.4)L
TLCO%*** (Available for *n* = 13)	57.4 (20.1)% (27.1–85.2)%
Baseline oxygen saturation (available for *n* = 15, *n* = 4 on supplemental oxygen (*n* = 1 4 L/min, *n* = 2 6 L/min, *n* = 1 10 L/min))	96(1.4)% (94–98)%

*FEV_1_ = Forced expiratory volume in the first second, **FVC = Forced vital capacity, ***TLCO = Transfer capacity of lung for carbon monoxide

**Table 3:** Health related quality of life (HRQoL), exercise capacity and physical activity outcomes

	Baseline Mean (SD)	Post PR Mean (SD)	Mean Difference (SD) 95% Confidence intervals	*P* –value
6MWT (*N* = 5)	361.5 (127.1) m	425.2 (136.1) m	63.7 (48.2) m [-3.8 to 123.6].	0.04
1 min STS	17.6 (3.0) (*N* = 11)	23.0 (6.3) (*N* = 10)	5.8 (4.6) [2.6 to 9.1]	0.003
K-BILD total (*N* = 15)	55.1 (7.4)	56.6 (7.9)	1.7 (6.5) [-1.7 to 5.3] *N* = 4 met the MCID	0.3
Psychological	54.1 (14.3)	58.8 (14.7)	4.7 (13.5) [-2.8 to 12.2] *N* = 6 met the MCID	0.2
Breathlessness & activities	39.7 (16.5)	39.9 (20.3)	-0.2 (14.2) [-7.7 to 8.0] *N* = 6 met the MCID	1.0
Chest symptoms	67.7 (15.4)	67.6 (18.2)	0.1 (11.6) [-6.5 to 6.3] *N* = 3 met the MCID	1.0
Mean daily time spent in Light PA	152 (69.4) mins (*N* = 16)	161.9 (88.7) mins (*N* = 14)	9.9 (38.8) mins [-12.3 to 30.9]	0.4
Mean daily time spend in MVPA	19.1 (18.6) mins (*N* = 16)	25.7 (28.3) mins (*N* = 14)	6.7 (15.5) mins [-2.1 to 15.1]	0.1
Mean daily Step count	3838 (2847) steps (*N* = 16)	4576 (3748) steps (*N* = 14)	738 (1916) steps [-419.3 to 1734.6]	0.2

SD- standard deviation, 6MWT- 6 min walk test, 1-minute STS = 1 min sit-to-stand, K-BILD = Kings Brief Interstitial Lung Disease, MVPA = Moderate to vigorous physical activity, MCID- minimal clinically important difference

## Discussion

The aim of this study to explore the feasibility of conducting a trial to explore effectiveness of VPR on objectively measured physical activity in people with IPF was achieved. We observed changes in physical activity among participants; unsurprisingly these were highly variable given the heterogeneity of the population. There are a number of important considerations in terms of recruitment and outcome measures to assess HRQoL in a future trial.

Importantly, participants in the current study demonstrated improvements in physical activity including daily step count and light physical activity despite the serious and progressive nature of their disease. There is little available literature on the amount and intensity of physical activity, for people with IPF; Hur et al. 2019 reported a minimal important difference (MID) of 12–69 min per week for MVPA, this was calculated over a six-month period with no intervention [25]. We observed an improvement of 47 min of MVPA per week which is line with this MID. There is no available guidance for light physical activity, there are only two studies to our knowledge that have reported on light PA levels in people with IPF [2, 26]. This is interesting given that there has been a shift in current literature towards promoting light physical activity in those with chronic respiratory disease [27]. We observed a mean increase of 10 min of light PA. It is unclear what the potential clinical impact of this is in an IPF population. Driver et al. have reported that an increase of 22 min of light physical activity per day can improve symptoms in those with COPD [27]. Furthermore, we observed a mean increase of 738 steps per day. Again, while we don’t have evidence to judge the potential impact of this improvement for people with IPF it is encouraging to see increased activity levels in this cohort with a progressive life limiting condition. More research exploring the impact of changes in physical activity on clinical outcomes in IPF is needed.

The recruitment rate for the IPF population in this study was 34%. This appears to be line with other research for PR in people with IPF (32–35%) [28, 29], however, the numbers screened and the reasons for non-participation are not fully detailed in these published works [30–32]. The primary reason for patients declining VPR in our study was lack of IT support. Despite this, those who did participate reported few problems with the technology aspect of the rehabilitation and any problems were quickly resolved [33]. The World Health Organisation expects digital technology to create a more equitable future for healthcare [34]; researchers, clinicians and policy makers should therefore strive to enable those who currently cannot access programmes due to lack of IT support. Finally, the results of this feasibility study enabled us to calculate a sample size for a fully powered trial to detect changes in physical activity before and after PR. Depending on the physical activity variable employed (light physical activity /step count/MVPA) a sample size is 22 (MVPA), 149 (light) or 1379 (step count) is required. The large standard deviations and wide confidence intervals in our results are noteworthy demonstrating the heterogeneous nature of our sample, which is reflected in the disease severity of participants. Strategies to enhance recruitment for a fully powered trial to explore changes in physical activity following PR in people with IPF would be needed for example offering a choice between remote and in-person rehabilitation and additional delivery sites. We observed no adverse events and high retention (94%) and adherence rate (90%).

We observed a small improvement in HRQoL. However, this improvement was only attributed to changes the psychological domain of the K-BILD, but did not meet the MCID for this domain [20]. The improvement in the psychological domain in the current study was reflected in the qualitative arm of this study where all participants expressed high levels of enjoyment and satisfaction with the programme despite some people experiencing a physical decline [33]. It is not clear if the K-BILD is the best tool to assess changes in HRQoL before and after PR, none of the studies included in the Cochrane review by Dowman et al. [9] used the K-BILD to measure changes in HRQoL. Future research should explore the sensitivity and responsiveness of the available measures of HRQoL measures in people with IPF before and after PR.

This novel research reports on objectively measured changes in physical activity in people with IPF following VPR. We have completed a checklist (Table 1) recently published by Iwakura et al., to promote higher standards of reporting with regard to accelerometer measured physical activity in people with IPF [24]. While this research provided us with a number of important insights into this population it is not without its limitations. We did not reach our target of 30 participants, it is not clear what impact this had on our findings, nonetheless important feasibility data relating to recruitment, retention, adherence and safety were gathered. This study was conducted during the COVID-19 pandemic, research has indicated reduced physical activity levels across populations during this time [35] and some participants in the current study also reported that reduced activity in the qualitative arm of the study [33]. It is therefore not clear if changes in physical activity would be different if data were collected under normal conditions. Furthermore, due to the COVID-19 restrictions two different measures of exercise capacity were employed in the current study, given the small sample size we are unable to discuss changes in exercise capacity in the context of the wider literature. There was also a lack of standardisation across the one-minute STS as participants were not instructed to use a standard chair height. Lastly, this study lacked both a control group in terms of individuals who did not receive VPR and a comparison against traditional centre based PR, future research should explore this.

## Conclusion

VPR can improve physical activity in people with IPF. A number of important feasibility issues included recruitment, retention, adherence and safety have been reported which are crucial for future research in this area. A fully powered trial is needed to determine the response of people with IPF to PR with regard to physical activity.

### Electronic supplementary material

Below is the link to the electronic supplementary material.

Supplementary Material 1

## Data Availability

Data are available on reasonable request from the corresponding author.

## Declarations

All methods were carried out in carried out in accordance with relevant guidelines and regulations. Research processes were carried in accordance with local ethical guidelines.
