# Supplementary material for: Changes in physical activity in people with idiopathic pulmonary fibrosis before and after virtual pulmonary rehabilitation: a feasibility study
Source: BMC Pulm Med. 2024 May 2;24:215. doi: 10.1186/s12890-024-03030-9 (PMC11064383; doi:10.1186/s12890-024-03030-9)
Supplement: Supplementary file 1 — Supplementary Material 1 [file 12890_2024_3030_MOESM1_ESM.docx]

COVID-19 restrictions Ireland

Restrictions varied throughout the study period from level 5 (October to December 2020 and December 2020- May 2021). Level 5 restrictions included for example no social gatherings, travel restricted to 5km from home except for work, people were advised to work from home where possible and all non-essential retail was closed (<https://www.gov.ie/en/press-release/066ce-ireland-placed-on-full-level-5-restrictions-of-the-plan-for-living-with-covid-19/>). There was a gradual reopening of society for example non-essential retail could initially provide click and collect and services by appointment, in May 2021 and restaurants and bars could provide outdoor dining in June 2021. The full timeline of restrictions can be viewed at <https://www.youtube.com/watch?v=BF6oLSwKMF0>
